# Achieving synchrony: bridging the gap between pharmaceutical companies and regulators on safety labeling updates

**DOI:** 10.3389/fdsfr.2024.1477516

**Published:** 2024-09-18

**Authors:** Tarek A. Hammad, Sasan Sabrdaran, Jean-Marie Heim

**Affiliations:** ^1^ Medical Safety of Marketed Products Development and Plasma-Derived Therapies, Patient Safety and Pharmacovigilance, Takeda Pharmaceuticals Inc., Cambridge, MA, United States; ^2^ Medical Safety of Marketed Products Development, Patient Safety and Pharmacovigilance, Takeda Pharmaceuticals Inc., Cambridge, MA, United States; ^3^ EU-QPPV Office, Patient Safety and Pharmacovigilance, Takeda Pharmaceuticals Inc., Cambridge, MA, United States

**Keywords:** drug safety, pharmacovigilance, safety labeling updates, regulatory alignment, synchrony challenges

## 1 Introduction

Safety labeling update is a critical component of pharmacovigilance, providing essential information to healthcare providers and patients about the potential risks associated with medications. Regulatory agencies such as the FDA and EMA, as well as many others, set stringent guidelines for safety labeling. These guidelines are designed to ensure that safety information is communicated clearly and effectively in a timely manner. The regulators require continuous monitoring of drug safety and mandate label updates when new risks are identified. Each regulatory body has its own set of regulations and procedures for updating safety labels, reflecting the unique healthcare landscape and legal framework within their jurisdiction.

Pharmaceutical companies are obligated to follow regulations and are responsible for continuously monitoring the safety of their products and updating safety labels as new information becomes available. This involves collecting and analyzing adverse event reports, reviewing data from various sources including clinical trials, preclinical data, post marketing surveillance, disease epidemiology, and literature. The process of identifying and confirming safety signals involves detailed assessment that might require further studies, sometimes leading to delays and negotiations. Once a potential safety concern is confirmed, companies evaluate the need for a label update. The internal processes for initiating and managing safety labeling updates are complex and require coordination across multiple departments, including pharmacovigilance, clinical science, and regulatory affairs, among others. Eventually, the information is added to the Company Core Data Sheet (CCDS), which represents the company’s position on the product.

In some instances, companies and regulators may not be aligned on the assessment of a safety finding. As a result, this finding will not be included in the CCDS but included in local labels, such as the Summary of Product Characteristics (SmPC) or U.S. Prescribing Information (USPI). These differences may be driven by variations in the timing of safety signal recognition, company-led updates, regulatory decision-making timelines, and differing interpretations of safety data between companies and regulatory bodies. They can also result from variations in the methodologies used for data analysis and causality assessment (Hammad et al., 2023a), as well as differing thresholds among regulatory agencies for determining the significance of a particular safety signal. Regulators also have access to extensive information about entire classes of drugs, which may include unpublished data, potentially class-wide safety concerns, and insights from their global pharmacovigilance activities. This broader perspective enables them to make decisions about safety labeling, incorporating data and considerations that may not be readily available to individual companies.

## 2 Challenges in alignment of safety labeling updates

The process of implementing safety labeling updates involves several steps, including the submission of supporting data, review by regulatory experts, negotiations between companies and regulators and public communication of the updates. This process may vary between different regulatory agencies, creating harmonizing challenges for pharmaceutical companies operating globally. Navigating multiple regulatory landscapes simultaneously involves addressing different regulatory requirements, expectations, and often lengthy review timelines, which can lead to commonly occurring discrepancies between the safety labels and related actions made by different agencies across or within various regions (Zeitoun et al., 2014). Moreover, an article by Dr. Woodcock discusses various sources of evidence that may trigger and support safety-related labeling updates for drugs in the market (Woodcock et al., 2011). The article highlights the role of post-marketing surveillance and real-world evidence in identifying new safety issues as well as the complexity of the healthcare systems and the challenges in managing safety labeling. This might explain potential misalignments with companies or differences in approach between regulatory bodies, especially if different regulators interpret the same data differently or prioritize various sources of evidence.

Regulatory agencies may vary with each other in their decisions, reflecting different risk tolerance and regulatory philosophies. Discrepancies in adverse event listings between the USPI and the SmPC have been well-documented. A study comparing labeling for 12 brand antidepressants and anticonvulsants drugs found that, on average, the USPI contained 77 more adverse drug reactions (ADRs) than the SmPC and the ADR profile was found to be inconsistently reported between the United States and Europe, for the same drug. On average, only 29% of ADR terms were reported in both paired documents, although, the observation of this degree of inconsistency in antidepressant and anticonvulsive drug may not be representative of other therapeutic areas (Cornelius et al., 2016). Further, a comparison of ADR information in the labels for oral formulations of atomoxetine, methylphenidate, and modafinil across Australia, Denmark, and the United States revealed substantial variations, highlighting the significant differences in safety labeling across regions. The authors suggested a need for global consistency in drug safety communication to ensure that prescribers and patients have uniform access to safety information, regardless of location (Aagaard and Hansen et al., 2013). Similarly, a study examining the labels of 40 separate drugs from different therapeutic groups marketed in both Denmark and the United States found notable inconsistencies in listing of ADRs; of the 4,003 ADRs identified, only 47% were consistent between the two countries. These findings underscore the need for further harmonization to improve consistency in publicly available drug information across borders (Eriksson et al., 2014).

In Europe, for older products with national authorizations, aligning safety labeling across multiple EU member states might be particularly challenging due to the separate regulatory pathways leading to discrepancies in SmPCs across member states. The centralized procedure, while more uniform, does not cover all products, especially older or off-patent drugs. A study examined the discrepancies in adverse events listed in the SmPCs for the same drugs that are nationally authorized within the EU. The research focused on SmPCs for 100 active substances and found that 41% of the included active substances exhibited discrepancies in the number of indications and contraindications listed (Gahr et al., 2022).

Additionally, the experience and perspectives of individual regulatory reviewers can further complicate the decision-making process. Differences in reviewers’ backgrounds and expertise can lead to varying interpretations and decisions regarding the same safety data, leading to different conclusions among reviewers even within the same agency. A field study found that regulatory medical assessors’ perceptions of drug benefits and risks are even influenced by their personality traits and gender (Beyer et al., 2015). The study findings highlight the interplay between medical situations, personality traits, and gender in decision-making. This variability can result in inconsistent regulatory responses in different regions, further complicating harmonization of labeling changes.

Regulators sometimes take a more conservative approach to safety labeling updates, driven by the “precautionary principle”. This principle leads regulators in some situations to err on the side of risk aversion to mitigate potential risks, as discussed by Hans-Georg Eichler in his 2013 publication (Eichler et al., 2013), especially when there is conflicting information about safety signals for drugs with multiple alternatives available. Widespread public concern or Congressional or Parliamentary inquiries regarding drug safety sometimes play a role in influencing regulatory agencies to adopt a more conservative approach^1^,^2^. By taking a cautious stance, regulatory agencies aim to ensure that all potential risks are thoroughly evaluated and communicated, thereby safeguarding public health and responding proactively to stakeholder concerns.

On the other hand, regulators might sometimes be hesitant to adopt safety labeling changes proposed by companies. A concern for regulators is the potential impact on patients, particularly in serious indications with high unmet needs. For instance, in cases involving life-saving medications, regulators may be wary of making changes based on insufficient or inconclusive data that would scare patients away from essential treatments. The concern is that premature, overly cautious safety labeling changes can lead to underutilization of effective drugs, potentially impacting public health, negatively.

Another significant factor is the risk of premature channeling, where patients might be directed to other drugs in the same class that appear safer but eventually are confirmed to carry the same risks. This was particularly noticeable with antidepressant drugs, where initial safety communication made for the sake of transparency about starting to investigate a safety signal with one drug, led to higher utilization of others in the same class. However, the risk was later confirmed for the entire class (Hammad et al., 2006; Pamer et al., 2010). In that regard, regulators sometimes face challenges in substantiating a class effect, often taking years to build a compelling case (Croteau et al., 2022). The difficulty in proving that all drugs in a particular class share the same risk profile, given the nuances of different mechanisms of action and extent of use, complicates the decision-making process and might contribute to misalignment with companies or even between regulators.

Additionally, regulators sometimes suspect that companies have legal motives for wanting to add ADRs to labels. This suspicion might lead to reluctance in accepting proposed changes, as regulators aim to avoid being seen as responding to litigation pressures rather than robust scientific evidence. This dynamic contributes to an environment where both parties may have differing priorities and interpretations of safety data. It is worth noting that, from our extensive experience working in several companies, legal motives are not a factor in the work dynamics of safety teams. Pharmacovigilance is centered on transparency, and pharmaceutical companies’ safety teams have a duty to inform and protect patients by timely reporting safety findings based on the assessment of the available evidence at any given time.

Discrepancies in safety labeling can create challenges for prescribers and patients, leading to uncertainty about a drug’s safety and inconsistent medical advice. These inconsistencies may undermine patient adherence and trust in medications, emphasizing the need for clear and harmonized safety information to ensure safe prescribing practices. Variations in safety labeling across regions can also result in disparities in the standard of care, with patients receiving different advice or treatments based on local labeling differences, which can lead to unequal access to optimal treatments and a lack of consistency in patient care. On a global scale, such discrepancies have broader public health implications, especially in an interconnected world where patients and healthcare providers frequently access information from multiple countries.

## 3 Commentary and future directions

This article illustrates the multifaceted nature of misalignment in safety labeling updates between pharmaceutical companies and regulatory agencies, emphasizing the need for robust pharmacovigilance practices, transparent dialogue, and flexible approaches to regulatory compliance. By understanding these dynamics, both regulators and pharmaceutical companies can work towards more effective and timely safety label updates, ultimately benefiting patients.

It is essential to recognize that both regulators and drug manufacturers play crucial roles in the identification of safety concerns (Croteau et al., 2022). Therefore, the initiation of updates of safety labeling by regulatory authorities should not automatically be construed as a negative reflection on a company’s safety or labeling governance, practices, or reputation. This collaborative effort between regulators and manufacturers is vital for effective use of the medicinal product while maintaining the highest standards of drug safety.

Given the complexity of the process, when investigating misalignment with regulatory agencies it is imperative to focus on a broader assessment of the opinion of several agencies on the same drug-event safety concern rather than limiting the assessment to involve a single regulatory agency at a time. Gaining deeper insights into the nuances of the reasoning underlying labeling updates can provide a more comprehensive perspective on the root causes of these misalignments. Additionally, it is important to look beyond the mere number of misalignments, as qualitative evaluation is vital to identify trends or clusters that may be present. For instance, in some situations, misalignments might involve the same drug, procedure, or safety team. Identifying these patterns or trends can facilitate targeted assessment, initiate constructive stakeholder discussions, and enhance benchmarking and labeling guidance. To further this objective, creating sessions to discuss noteworthy scenarios involving misalignment can be beneficial. These discussions, along with providing (re)training in established forums, can foster a culture of continuous improvement and proactive engagement with regulatory authorities. By focusing on these broader strategies, the industry can better navigate the intricacies of safety labeling updates and ensure that both regulatory and manufacturer perspectives are aligned, to the extent possible, for the ultimate benefit of patients.

To improve alignment, companies should adopt best practices such as establishing robust pharmacovigilance systems, enhancing communication with different departments within the company and with regulators, and leveraging digital tools for efficient data management. The field of pharmacovigilance is evolving with advancements in technology and data analytics and the implementation of evidence-based medicine principles (Hammad et al., 2024). This trend holds the potential to transform the alignment process for safety labeling updates possibly minimizing interpersonal and interregional interpretational differences. Emerging trends such as the use of artificial intelligence (AI) for signal detection and risk assessment might help enhance the accuracy and efficiency of pharmacovigilance activities (Hammad et al., 2023b) through identifying safety signals more quickly and accurately, facilitating the prompt initiation of label changes. While AI alone does not directly solve the problem of misalignment, we believe it can play a supportive role. AI can help identify discrepancies in safety labeling across different regions or products more rapidly, allowing for quicker detection of inconsistencies and perhaps recommend updates where discrepancies exist. Furthermore, AI-driven analytics can provide insights into why certain safety updates are misaligned, helping companies and regulators understand the underlying causes and work toward harmonization. Additionally, AI can be used to suggest harmonized language for safety labels based on global data analysis, helping companies and regulators adopt a more unified approach.

Regulators can also contribute to improved alignment by providing clearer guidance on safety labeling updates, expediting review processes to minimize delays and enhancing transparency about the reasoning behind decision making. Potential regulatory changes, such as an effort to harmonize safety labeling requirements across regions, could also improve alignment by reducing the need for region-specific submissions and approvals. This would streamline the alignment process and ensure that safety labels are consistent globally. The future of global harmonization of safety labels, which require significant collaboration and coordination among regulatory bodies, holds promise but also presents substantial challenges that must be addressed to ensure that safety information is consistently accurate and reliable worldwide.

## Footnotes

1 Adams, B. EMA under fire from European Parliament. Pharmafile website [online], http://www.pharmafile.com/news/156464. accessed 23 June 2024.

2 United States Senate website [online]. https://www.finance.senate.gov/chairmans-news/grassley-questions-fda-about-information-on-antidepressants-suicide. Accessed 24 June 2024.
